# Gas therapy strategies for depression and schizophrenia: A review

**DOI:** 10.1097/MD.0000000000036156

**Published:** 2023-11-17

**Authors:** Xun Tao, Xiaoxuan Zhu, Yang Liu, Ling Wang, Dan Wang, Lin Sun, Changjiang Li, Bo Lian, Yingshuai Wang, Feng Chen

**Affiliations:** a School of Clinical Medicine, Weifang Medical University, Weifang, P. R. China; b School of Psychology, Weifang Medical University, Weifang Shandong, P. R. China; c Clinical Competency Training Center, Medical Experiment and Training Center, Weifang Medical University, Weifang Shandong, P. R. China; d Department of Physical Education, School of Foundation Medical, Weifang Medical University, Weifang, P. R. China; e Department of Bioscience and Technology, Weifang Medical University, Weifang, Shandong, P. R. China; f School of Practical Teaching Management Department, Weifang Medical University, Weifang Shandong, P. R. China.

**Keywords:** carbon monoxide, depression, hydrogen, hydrogen sulfide, nitric oxide, schizophrenia

## Abstract

Depression and schizophrenia are 2 serious mental disorders. Their effective treatment is an urgent medical and social problem at present. Drug treatment is the basic measure to improve mental disorders, especially serious mental disorders. However, the side effects of traditional antipsychotic drugs cannot be avoided. Surprisingly, in recent years, it has been found that nitric oxide (NO), carbon monoxide (CO), hydrogen sulfide (H_2_S) and hydrogen (H_2_) can regulate corresponding signal pathways to treat mental diseases in animal models. More importantly, as gas signal molecules, they will not bring toxicity and side effects after metabolism. Therefore, in this review, we analyzed the effects of gas on depression and schizophrenia through endogenous gas generation and external gas delivery strategies in some animal models. Endogenous gas generation strategy: summarized the therapeutic mechanism of gas signaling molecules on depression and schizophrenia, and listed the main ways to inhibit or stimulate gas generation. External gas delivery strategy: The common external stimuli-responsive gasotransmitter prodrugs and some study of these prodrugs in the treatment of depression and schizophrenia are summarized. We also analyzed the prospects of nano-gas carrier in the treatment of depression and schizophrenia. Through this review, we hope to provide guidance for treating depression and schizophrenia by regulating relevant gas signal pathways, and provide reference for developing safe and effective drugs for treating mental disorders by summarizing exogenous gas drugs.

## 1. Introduction

Depression refers to a kind of mood disorder caused by a variety of reasons and characterized by significant and persistent depressive symptoms. Anxiety often coexists with depression and becomes one of the main symptoms of depression. Cognitive dysfunction is also an important clinical feature of depression. At present, the commonly used oral antidepressants include monoamine oxidase inhibitor, tricyclic antidepressant, selective serotonin reuptake inhibitor, serotonin norepinephrine reuptake inhibitor and other atypical antidepressants. These antidepressants have many side effects, such as cholinergic adverse reactions, withdrawal syndrome, metabolic abnormalities, etc.^[1]^ Therefore, we should look for more effective and no side effects of depression drugs.

Patients with schizophrenia exhibit severe psychotic symptoms of 3 different types: cognitive dysfunction (such as attention, executive functioning, and memory issues), negative symptoms (such as asociality, anhedonia, and avolition), and positive symptoms (such as hallucinations, delusions, disordered thought processes, and catatonic behavior).^[2]^ At present, antipsychotic drugs are mainly divided into the first generation, the second generation and the third generation of antipsychotic drugs, and all of these drugs have serious adverse reactions.^[3]^ First generations (chlorpromazine and pimozide) are dopamine D2 antagonists, and the side effects of these drugs are mainly extrapyramidal symptoms.^[4]^ Second generations (clozapine and olanzapine) are multitargeted antipsychotics, and these drugs act on dopamine D1, D2, D3, and D4, the adrenergic alpha1 and alpha2, serotonin 5HT2A and 5HT2C, histamine and muscarinic receptors. The side effects of these drugs are mainly related to weight gain and metabolic syndrome.^[5,6]^ Therefore, new treatment strategies are also urgently needed for anti-schizophrenia.

Endogenous gas generation strategy provides new ideas for the treatment of depression and schizophrenia, and the strategy is subdivided into inhibition of gas generation and stimulation of gas generation. Nitric oxide (NO) modulates the release of some classical neurotransmitters and the onset of synaptic plasticity.^[7,8]^ NO molecules cross the synaptic gap into presynaptic neurons, where soluble guanyl cyclase (sGC) is activated and produces cyclic GMP (cGMP), thereby affecting the neurotransmitter release mechanism and regulating the opening of ion channels, thus influencing the pathogenesis of depression and schizophrenia.^[9]^ The facilitation of gas signaling molecules is mainly associated with the production of carbon monoxide (CO). CO can be produced by oxidative catabolism through injection of heme oxygenase (HO).^[10]^ CO has a similar affinity to NO-bound sGC, but CO-sGC is 25 to 50 times less active than NO-sGC.^[11]^ This paper describes research on gas therapy for depression and schizophrenia using the endogenous gas production strategies of NO and CO as examples.

**Figure 1.**
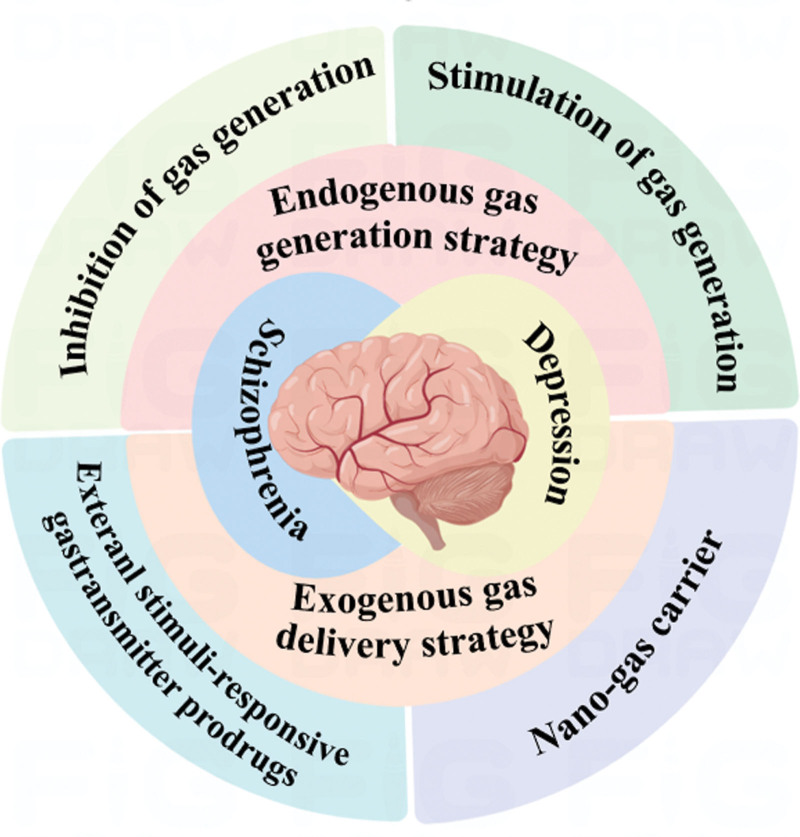
A number of drugs can affect depression and schizophrenia from both endogenous and exogenous pathways by either facilitating the production of gases or inhibiting the production of gases.

External gas delivery strategy also provides new ideas for the treatment of depression and schizophrenia. External stimuli-responsive gasotransmitter prodrugs are mainly facilitative gas donors. In addition, this paper also looks forward to the application of nano-gas carriers. These gas prodrugs mainly involve NO, CO, hydrogen sulfide (H_2_S), and hydrogen (H_2_). The targets of H_2_S for treating depression and schizophrenia are mainly distributed in N-methyl-D-aspartate (NMDA) and γ-aminobutyric acid (GABA) receptors.^[12,13]^ H_2_S can be neuroprotective through antioxidant enzymes and anti-inflammatory effects acted as a neuroprotective agent.^[14,15]^ In addition, high expression of oxidative stress biomarkers such as malondialdehyde is expressed in depressed patients, whereas hydrogen endogenous antioxidant enzymes are produced.^[16,17]^ Pro-inflammatory cytokines are increased in depressed patients, and hydrogen can inhibit the production of pro-inflammatory cytokines, so hydrogen can treat depression.^[18,19]^ Therefore, this paper summarizes the therapeutic effects of major gases on depression and schizophrenia through 2 strategies: endogenous gas production and exogenous gas supply strategies (Fig. 1).

## 2. Endogenous gas generation strategy

Endogenous gas treatment strategies mainly include both inhibition of gas generation and stimulation of gas generation, and this section summarizes endogenous gas treatment strategies for the treatment of depression and schizophrenia, using NO and CO as examples, respectively (Table 1).

**Table 1:** Endogenous and exogenous substances in paradigms of depression and schizophrenia.

Strategy	Type	Substance	Disease	References
Endogenous	Inhibition	AG	Schizophrenia	^[27]^
		L-NOARG	Schizophrenia	^[26]^
		AG	Depression	^[24]^
		7-NI	Depression	^[22]^
	Stimulation	Heme-lysinate	Depression	^[32]^
External	External stimuli-responsive gasotransmitter prodrugs	SNP	Depression	^[34]^
		SNP	Schizophrenia	^[35]^
		Molsidomine	Depression	^[46]^
		Molsidomine	Schizophrenia	^[47]^
		DETA/NONOate	Depression	^[52]^
		CORM-2	Depression	^[53]^
		CORM-3	Depression	^[55]^
		CO-rich saline or CO gas	Depression	^[56]^
		NaHS	Depression	^[58]^
		Na_2_S	Depression	^[64]^
		DADS	Depression	^[67]^
		Hydrogen-Rich Water	Depression	^[71]^
	Nano-gas carrier	CORM-2-SLN	Depression	^[54]^

These gas-related drugs can affect the development of depression and schizophrenia by either stimulating the production of gas or inhibiting its production. 7-NI = 7-Nitroindole, AG = aminoguanidine, CO = carbon monoxide, CORM-2 = CO-releasing molecule 2, DADS = diallyl disulfide, L-NOARG = L-NG-nitro arginine, SNP = sodium nitroprusside.

### 2.1. Inhibition of gas generation

Nitric oxide is produced via l-arginine by nitric oxide synthase (NOS), which exists in 3 distinct isoforms: neuronal nNOS (NOS 1), inducible iNOS (NOS 2), and endothelial eNOS (NOS 3). NOS inhibitors are typical substances that inhibit NO production. The enzyme NOS, which is reliant on calcium (Ca^2+^) and calmoduline, converts L-arginine into L-citrulline to produce NO.^[20]^ 7-Nitroindole (7-NI) is a selective neuronal nitric oxide synthase (nNOS) inhibitor that treats depression by competing with L-arginine for the binding site of L-arginine on NOS.^[21]^ 7-NI exerts similar antidepressant effects in the rat experiments.^[22]^ NO affects neurotransmitter release by synthesizing cGMP through sGC. Aminoguanidine (AG) is a hydrazine derivative that inhibits inducible NO synthase (iNOS) and thus reduces cGMP production.^[23]^ Intracerebroventricular infusion of AG can block depression-like behaviors resulting from chronic unpredictable stress paradigms.^[24]^

NMDA receptor activation is crucial in the production of NO.^[25]^ N(omega)-nitro-L-arginine methyl ester (L-NAME) and L-NG-nitro arginine (L-NOARG) are nonselective inhibitors of NOS. L-NAME prevents phencyclidine (PCP)-induced hyperkinesia and attention deficit in rats. Attention impairment induced by PCP is also reduced in rats by pretreatment with L-NOARG.^[26]^ The selective iNOS inhibitors AG and epigallocatechin gallate reduced PCP-induced hyperkinesia and reversed dizocilpine (MK-801-induced) psychotomimetic symptoms in rats, and both AG and epigallocatechin gallate normalized MK-801-stimulated extracellular glutamate levels in the medial prefrontal cortex.^[27]^ This suggests that modulation of NMDA receptors with NOS inhibitors may be a target for the treatment of schizophrenia cognitive disorders. 7-NI reduces the psychotomimetic effects of apomorphine in mice.^[28]^ This suggests that 7-NI can also treat schizophrenia and cognitive disorders associated with dopaminergic dysfunction. Cognitive impairment is an important manifestation of depression, and depression is also associated with abnormalities in MADA receptors as well as in the dopamine system. Therefore, this also implies that NOS inhibitors can alleviate depression.

Studies related to NO inhibitors in depression have shown conflicting results. For example, anxiety-like effects following NOS inhibition were found in the rat elevated plus maze with systemic^[29]^ and central^[30]^ administration of L-NOARG or L-NAME, and in the mouse light-dark box test test with systemic administration of the selective neuronal NOS inhibitor 7-NI.^[31]^ Based on the current studies, this may be related to the type, concentration, and mode of injection of NOS inhibitors. Therefore, when using nitric oxide synthase inhibitors to treat depression, attention should be paid to the injection method and dosage, among other things.

### 2.2. Stimulation of gas generation

The main endogenous gas-promoting signaling molecule is hemoglobin. The locus coeruleus (LC)^[32]^ is a part of the central nervous system that is intimately involved in the regulation of emotional behavior and stress as well as fear and depression. The LC expresses HO, which catalyzes the conversion of hemoglobin to bilirubin, resulting in the production of the endogenous gas mediators CO and Fe^2+^. CO and NO have similar properties and activate the sGC, resulting in a complex regulation of cGMP. Microinjection of heme (a substrate of HO enzyme) in LC to promote CO production can alleviate anxiety and depression.^[32]^ Furthermore, the anxiolytic effect was suppressed after microinjection of heme into the LC and microinjection of guanyl cyclase (an sGC inhibitor) into the lateral ventricle. Thus, it can be said that CO in the rat LC is produced by the HO pathway and acts through cGMP, which functions similarly to antidepressants. However, too much CO can also lead to side effects such as CO poisoning, so the optimal concentration of CO for treating depression still needs to be further explored.

## 3. External gas delivery strategy

In addition to endogenous gas intervention, the supplement of exogenous NO, CO, H_2_S and H_2_ gas is effective in the treatment of depression and schizophrenia. The following is a review of typical external stimuli-responsive gasotransmitter prodrugs releasing NO, CO, H_2_S and H_2_ in the treatment of depression and schizophrenia. The paper also looks at the future of nano-gas carriers for the treatment of psychiatric disorders.

### 3.1. External stimuli-responsive gasotransmitter prodrugs

**Figure 2.**
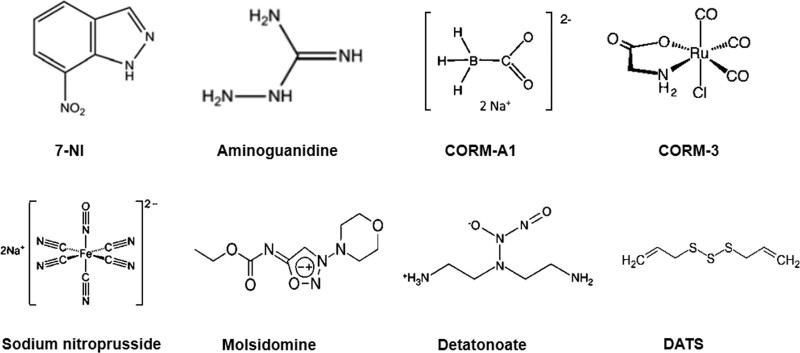
Chemical structures of some of the main substances that regulate gas production through endogenous or external strategies. 7-NI = 7-Nitroindole, DATS = diallyl trisulfide.

Exogenous NO donors can be used to treat not only depression but also schizophrenia. Common exogenous NO donors include Sodium nitroprusside (SNP), Molsidomine, and DETA/NONOate (Fig. 2). SNP is a member of the prussides family and an NO donor. It is composed of an iron core, 5 cyanide ion molecules and a nitrosoammonium ion (NO^+^) molecule.^[33]^ Although SNP can alleviate the symptoms of depression, acute administration of SNP 30 minutes (but not 60 minutes) before the test induces anxiety-like behavior in rats.^[34]^ It is suggested that the therapeutic effect of SNP on depression is related to drug dose and time of administration, and that there is a limited therapeutic window in this animal model of depressive tendencies. The NO donor SNP is also expected to be a new drug for the treatment of schizophrenia and neurocognitive disorders. SNP is a fast-acting vasodilator that increases cerebral perfusion and helps to treat the typical symptoms of cerebral underperfusion in patients with schizophrenia.^[35]^ According to a body of evidence,^[36,37]^ SNP may correct schizophrenia-like symptoms caused by the pharmacological effects of the glutamatergic and DAergic systems. The NMDA-nNOS-cGMP pathway is impaired in schizophrenia, and SNP treats schizophrenia by complementing the NMDA-nNOS-cGMP pathway.^[38]^ The D1/D2 mixed receptor agonist apomorphine dose-dependently impairs short-term recognition memory in rats, but SNP reduces apomorphine amnesia by blocking the stimulation of D1/D2 mixed receptors by apomorphine thereby improving cognitive impairment.^[39]^ Schizophrenia is associated with oxidative stress, while apomorphine^[40]^ and ketamine^[41]^ can increase oxidative stress in the mouse brain thereby causing neurocognitive impairment. However, SNP has been found to have strong antioxidant activity in many animal models of neurodegenerative diseases,^[42]^ suggesting that SNP may treat schizophrenia and cognitive disorders through antioxidants.

Moxidomine is one of the most bioavailable and longest acting NO donors.^[43]^ Studies have shown that it can cross the blood-brain barrier (BBB)^[44]^ and enhance its permeability.^[45]^ Its anxiolytic effect is similar to that of the benzodiazepine anxiolytic diazepam.^[46]^ Molsidomine can also alleviate some symptoms of schizophrenia. Molsidomine has been shown to have anticonvulsant effects in rats^[47]^ and to potentiate the anticonvulsant effects of riluzole and valproic acid (two independent NMDA antagonists) in mice.^[48]^ In many animal models, molsidomine works well against memory deficits, suggesting that it can improve cognitive impairment.^[49,50]^ Both patients with depression and schizophrenia are likely to have cognitive impairment including memory impairment, so this suggests that molsidomine holds promise for treating both depression and schizophrenia.

DETA/NONOate is a long half-life NO donor,^[51]^ releasing 2 molecules of NO per molecule and significantly promoting proliferation, survival, migration and differentiation of hippocampal neural precursor cells. Treatment of depression with pure DETA/NONOate could produce similar antidepressant effects by promoting hippocampal neurogenesis.^[52]^

External CO donors can treat depression. The anxiolytic effect of LC in rats can be promoted by intraperitoneal injection of CO-releasing molecule 2 (CORM-2) by increasing the activity of heme oxygenase 1 (HO-1).^[53]^ However, its half-life for CO release is very short. To overcome this difficulty, CORM-2 solid lipid nanoparticles (CORM-2-SLN) were fabricated and their improvement in preventing blood-spinal cord barrier (BSCB) disruption and endothelial cell death after spinal cord injury was investigated.^[54]^ CORM-3 treatment increased brain circulation, promoted amygdala neuron survival, and significantly reduced anxiety-like behavior in traumatic brain injury rats.^[55]^ Administration of saline enriched with CO or CO gas has similar effects as antidepressants and anxiety relievers.^[56]^ Currently, there are few studies on the treatment of depression with exogenous CO donors, but this is a promising direction of research that could guide the work on gas medication for depression.

Exteranl H_2_S donors are also effective in improving depressive symptoms and schizophrenia as well as cognitive impairment. In a model of homocysteine-induced cognitive impairment, a decrease in H_2_S synthase, an increase in NMDA receptor expression and an increase in synaptosomal Ca^2+^ were observed. Exogenous supplementation with sodium thioredoxin normalizes NMDA receptor and calcium ion concentrations, thereby ameliorating cognitive impairment.^[57]^ It has also been shown that H_2_S enhances synaptic plasticity by maintaining NMDA receptor and calcium ion concentrations, making long-term potentiation in the hippocampal nucleus easier to evoke thereby improving mental status.^[58]^ In other models of homocysteine-induced cognitive impairment, NaHS improves the expression of nuclear factor E2-related factor 2 (Nrf2) and other antioxidants to prevent cognitive decline.^[59]^ NaHS also reduce the level of reactive aldehydes by increasing aldehyde dehydrogenase 2 expression in the hippocampus and decreasing glutathione levels to treat cognitive disorders.^[60]^ In addition, exogenous administration of NaHS has been shown to improve cognitive decline induced by bilateral intraventricular lipopolysaccharide by reducing TNF-α levels, decreasing TNF receptor expression, and inhibiting nuclear factor kB activation in the hippocampal region,^[61]^ suggesting that H_2_S may improve cognitive symptoms by reducing the development of neuroinflammation. H_2_S-releasing drugs may also have a beneficial effect on learning memory with beneficial effects.^[62]^ Low doses of hydrogen sulfide can be used to improve cognitive impairment, but high doses of H_2_S are extremely neurotoxic.^[63]^ Cognitive impairment is the most common core symptom of schizophrenia and an important clinical symptom of depression. These studies suggest that H_2_S can treat cognitive impairment in many animal models, which provides an important foundation for research on H_2_S treatment of depression and schizophrenia.

In terms of anxiety and depression, H_2_S may also treat depression by modulating GABA, the main target of depression-related treatments such as benzodiazepines, where stimulation of GABAA receptors is associated with anxiolytic activity, while its inhibition may trigger anxiety responses. In the central nervous system, H_2_S is involved in several processes that promote long-term potentiation and upregulation of GABAB receptors in the hippocampus. Increased synaptic GABA concentrations promote silencing of postsynaptic neurons, which may support the antidepressant effect of H_2_S. Fast exogenous H_2_S donors, such as NaHS^[58]^ and Na_2_S,^[64]^ have been shown in previous studies to have anxiolytic or antidepressant effects and to alleviate anxiety or depressive behaviors associated with diabetes.^[65]^ Chen suggested that this is due to the function of H_2_S in regulating oxidative stress and neuroplasticity by reducing reactive oxygen species and thus treating depression.^[66]^ In addition, diallyl disulfide, a component of garlic and a donor of H_2_S, has antidepressant effects in mice with depressive behavior caused by congenital mitral stenosis.^[67]^ Currently, there are few known studies on the treatment of depression with exogenous H_2_S donors, but some studies suggest that this is an effective treatment and therefore it is possible to develop H_2_S drugs and use them in clinical trials for the treatment of depression.

External H_2_ donor can treat depression. Molecular H_2_ is a natural element existing in nature and biology, which can reduce inflammatory reaction and excessive oxidative stress. H_2_ can cross the BBB and penetrate the cell membrane, producing neuroprotective effects in the brain, and there is lack of evidence to show that the adverse side effects or tolerance development caused by repeated treatment of H_2_. The antioxidant effect of H_2_ mainly depends on its ability to stimulate the transfer of Nrf2 transcription factor from cytoplasm to nucleus, including the transcription of several genes, such as superoxide dismutase, HO-1 and NAD (P) H: quinone oxidoreductase 1 (NQO1).^[68,69]^ At present, there are several methods to apply H_2_, including inhaling hydrogen (H_2_ gas), drinking H_2_ dissolved water (H_2_ water) and injecting H_2_ dissolved brine (hydrogen-rich brine).^[70]^ In recent years, hydrogen-rich water has become a promising therapeutic strategy for preventing and intervening stress-related diseases due to its antioxidant and anti-inflammatory properties.

The strong relationship between oxidative stress and inflammation and depression and/or anxiety disorders has been revealed. The expression of oxidative and inflammatory biomarkers in specific brain areas of depression patients has increased, while H_2_ has the ability to reduce inflammation by reducing IL-6, IL-1β, IL-12 and TNF-α.^[71,72]^ Repeated inhalation of H_2_ and oxygen mixture [67%: 33% (V/V)] can significantly reduce the depression-like and anxiety-like behavior induced by acute and chronic stress in mice through tail suspension test, forced swimming test, novel inhibition feeding test and open field test. The injection of HRW into the abdominal cavity of male mice with chronic inflammatory pain can not only alleviate the inflammatory pain, but also inhibited the behaviors related to depression-like and anxiety-like behaviors.^[71]^

Repeated inhalation of high concentrations of H_2_ gas relieves stress-induced depression and anxiety in mice.^[73]^ Saline enriched with H_2_ had similar anxiolytic effects in morphine withdrawal mice.^[74]^ These studies suggest that hydrogen gas can treat depression. However, research on H_2_ in schizophrenia is lacking, with only a few studies on the treatment of cognitive disorders. Inhalation of 2% H_2_, similar to H_2_S, also alleviates blood-brain barrier damage and cognitive dysfunction in septic mice via an Nrf2-dependent pathway.^[75]^ Oxidative stress damage to neurons in the CA1 region of the hippocampus leads to cognitive dysfunction. H_2_ inhalation attenuates transient whole brain ischemia-induced cognitive dysfunction by attenuating neuronal oxidative stress in the CA1 region.^[76]^ Hydrogen-rich saline attenuates isoflurane-induced caspase-3 activation and cognitive impairment by inhibiting isoflurane-induced oxidative stress, mitochondrial dysfunction, and reduced ATP levels.^[77]^ These studies suggest that H_2_ can alleviate cognitive impairment and has important research implications for the treatment of schizophrenia since cognitive impairment is a core symptom of schizophrenia.

### 3.2. Nano-gas carrier

**Figure 3.**
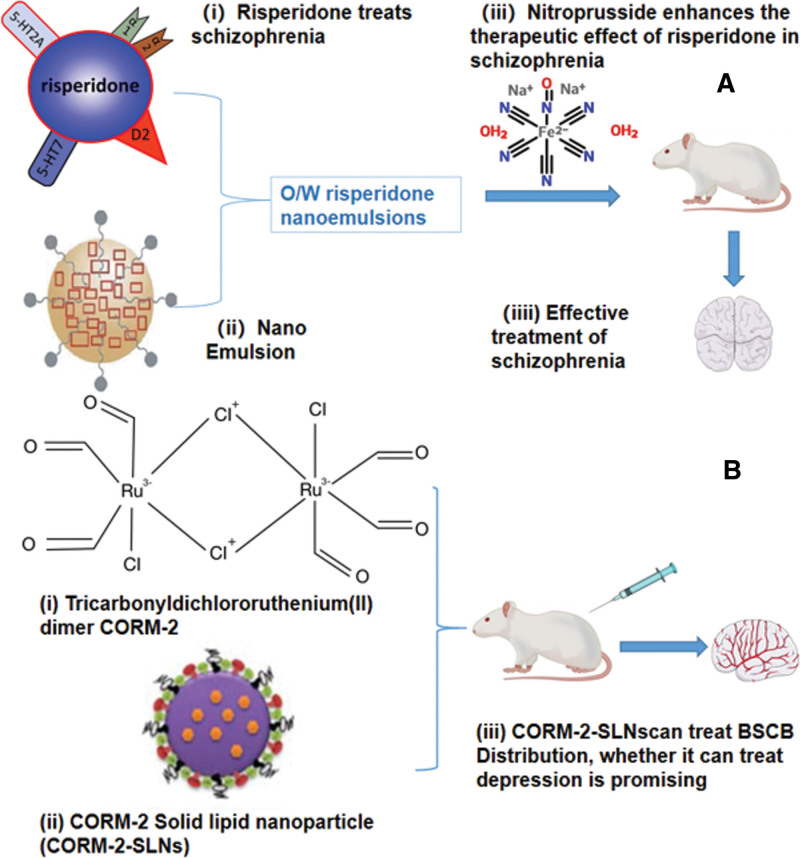
(A) Risperidone exerts its lasting effect by synthesizing O/W nano lotion with nano emulsion. Sodium nitroprusside can enhance the antipsychotic effect of risperidone and effectively treat schizophrenia; (B) CORM-2-SLN can easily cross the blood-spinal cord barrier (BSCB) or blood-brain barrier (BBB), and can release CO slowly. The combination of CORM-2 and nanoparticles to make new drugs can treat diseases more effectively.

Current medications for depression and schizophrenia have many disabling side effects, many of which are lipophilic, making them difficult to form at high intensities. Combining them with nanomaterials can increase their solubility, prevent their degradation, and increase their brain delivery, which can overcome the current therapeutic challenges. And since gaseous neurotransmitters improve depression and schizophrenia, combining gaseous neurotransmitters with nanomaterials to make exogenous gas-delivery donors may be a promising therapeutic strategy (Fig. 3). For example, Risperidone exerts its lasting effect by synthesizing O/W nano lotion with nano emulsion. CORM-2-SLN can easily cross the BSCB or BBB, and can release CO slowly.

The release of CO by intraperitoneal administration of CORM-2 molecules can promote the anti-anxiety effect and increase HO-1 activity in rat LC. However, its CO emission half-life is very short, so CORM-2-SLN have been developed. Because of their amphiphilic physicochemical properties, these CORM-2-SLN can easily cross the BSCB or the BBB, and can release CO slowly.^[54]^ Therefore, the combination of CORM and nanoparticles to make new drugs can more effectively treat diseases, and whether it can be used to treat depression deserves further study. CORM-2 has been proved to have an improvement effect on depression, so the study of CORM-2-SLN will promote the development of drugs to treat depression.

Risperidone is one of the oldest second-generation antipsychotic drugs, used as an inhibitor of dopaminergic D2 and serotonergic 5-HT2A receptors for the treatment of schizophrenia.^[78]^ However, oral administration of risperidone is associated with side effects such as tremors and slow onset of action. To address this problem, Dordevic et al developed O/W nanoemulsions for parenteral administration.^[78,79]^ The nanoemulsions are almost 6 times more soluble in water than risperidone. The formulation does have a longer lasting antipsychotic effect with fewer side effects compared to the drug solution. As mentioned above, SNP is a typical exogenous NO donor. It is reported that SNP can enhance the antipsychotic effect of risperidone.^[80]^ Therefore, the possibility of SNP and risperidone in combination with nanoemulsion in the treatment of schizophrenia can be further studied, and it is of great significance for the production of anti-schizophrenia gaseous nanodrugs. At present, there are very few studies on nanocarrier gas for the treatment of psychiatric disorders, but nano-gas carrier can treat psychiatric disorders in an efficient and energy-saving way, and the development of nano-gas carrier drugs is very promising.

## 4. Conclusion

Depression and schizophrenia are 2 common mental illnesses. In this paper, we review the mechanisms of action and therapeutic prospects of NO, CO, H_2_S and H_2_ in depression and schizophrenia, mainly from both endogenous and exogenous aspects. At normal levels, they may have a preventive effect against psychiatric disorders, but an increase or decrease of these gaseous signaling molecules will lead to pathological conditions of neuropsychiatric disorders. Therefore, maintaining optimal levels of these gastransmitters may be beneficial in the treatment of neuropsychiatric disorders. Gas therapy provides new ideas for the treatment of depression and schizophrenia, and research on nano-gas carrier treatment for mental illness is in its infancy, providing guidance for the further development of gas therapy drugs for the treatment of depression and schizophrenia in the future.

## Acknowledgments

This work was supported by National Natural Science Foundation of China (NSFC) (82101588). XT proposed the ideas and wrote the article, XZ and YL revised the article, LW and DW provided funding, LS and CL provided pictures, BL created the tables, and YW and FC guided the writing and layout of the article.

## Author contributions

**Data curation:** Xiaoxuan Zhu, Lin Sun.

**Methodology:** Yang Liu, Changjiang Li.

**Project administration:** Ling Wang.

**Resources:** Dan Wang, Feng Chen.

**Validation:** Bo Lian.

**Writing – original draft:** Xun Tao, Yingshuai Wang.

**Writing – review & editing:** Xun Tao.
